# 4-Amino­phenyl naphthalene-1-sulfonate

**DOI:** 10.1107/S1600536808014438

**Published:** 2008-05-21

**Authors:** Jasmine P. Vennila, R. Thilagavathi, R. Kavipriya, Helen P. Kavitha, V. Manivannan

**Affiliations:** a Department of Physics, Panimalar Institute of Technology, Chennai 600 095, India; b Department of Chemistry, SRM University, Ramapuram, Chennai 600 089, India; c Department of Physics, Presidency College, Chennai 600 005, India

## Abstract

In the title compound, C_16_H_13_NO_3_S, the plane of the amino­benzene ring makes a dihedral angle of 61.04 (6)° with the naphthalene ring system. Both ring systems form weak intra­molecular C—H⋯O hydrogen bonds with the sulfonate group. In the crystal structure, weak inter­molecular N—H⋯O hydrogen bonds and a C—H⋯π inter­action are observed.

## Related literature

For biological activity, see: Yachi *et al.* (1989 ▶). For the structures of closely related compounds, see: Manivannan *et al.* (2005*a* ▶,*b* ▶).
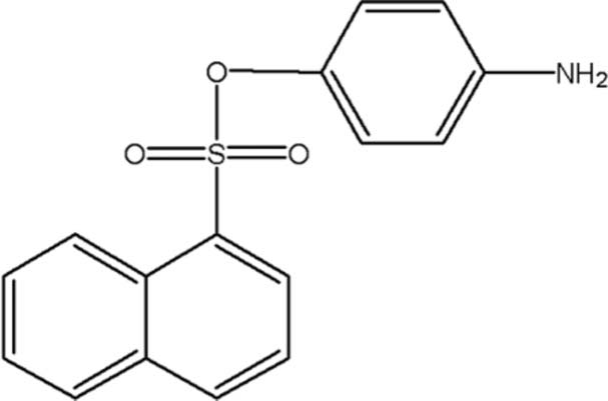


## Experimental

### 

#### Crystal data

C_16_H_13_NO_3_S
*M* *_r_* = 299.33
Orthorhombic, 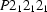

*a* = 7.0456 (3) Å
*b* = 12.4789 (6) Å
*c* = 15.8064 (8) Å
*V* = 1389.72 (11) Å^3^
*Z* = 4
Mo *K*α radiation
μ = 0.24 mm^−1^
*T* = 295 (2) K
0.36 × 0.16 × 0.16 mm

#### Data collection

Bruker Kappa APEXII diffractometer
Absorption correction: multi-scan (**SADABS**; Sheldrick, 1996 ▶) *T*_min_ = 0.918, *T*_max_ = 0.962
19808 measured reflections
4629 independent reflections
3267 reflections with *I* > 2σ(*I*)
*R*_int_ = 0.030

#### Refinement

*R*[*F* ^2^ > 2σ(*F* ^2^)] = 0.051
*wR*(*F* ^2^) = 0.164
*S* = 1.03
4629 reflections
190 parameters
2 restraints
H-atom parameters constrained
Δρ_max_ = 0.44 e Å^−3^
Δρ_min_ = −0.37 e Å^−3^
Absolute structure: Flack (1983 ▶), 1972 Friedel pairs
Flack parameter: −0.01 (10)

### 

Data collection: *APEX2* (Bruker, 2004 ▶); cell refinement: *APEX2*; data reduction: *APEX2*; program(s) used to solve structure: *SHELXS97* (Sheldrick, 2008 ▶); program(s) used to refine structure: *SHELXL97* (Sheldrick, 2008 ▶); molecular graphics: *PLATON* (Spek, 2003 ▶); software used to prepare material for publication: *SHELXL97*.

## Supplementary Material

Crystal structure: contains datablocks I, global. DOI: 10.1107/S1600536808014438/is2293sup1.cif

Structure factors: contains datablocks I. DOI: 10.1107/S1600536808014438/is2293Isup2.hkl

Additional supplementary materials:  crystallographic information; 3D view; checkCIF report

## Figures and Tables

**Table 1:** Hydrogen-bond geometry (Å, °)

*D*—H⋯*A*	*D*—H	H⋯*A*	*D*⋯*A*	*D*—H⋯*A*
C6—H6⋯O3	0.93	2.52	3.038 (3)	116
C8—H8⋯O3	0.93	2.38	2.804 (4)	108
C15—H15⋯O2	0.93	2.44	3.071 (4)	125
N1—H1*B*⋯O1^i^	0.86	2.05	2.909 (3)	173
N1—H1*A*⋯O3^ii^	0.86	1.94	2.773 (3)	162
C6—H6⋯*Cg*^iii^	0.93	2.84	3.380 (3)	107

Symmetry codes: (i) 
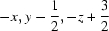
; (ii) 
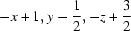
; (iii) 
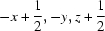
. *Cg *is the centroid of the C11–C16 ring.

**Fig. 1.**
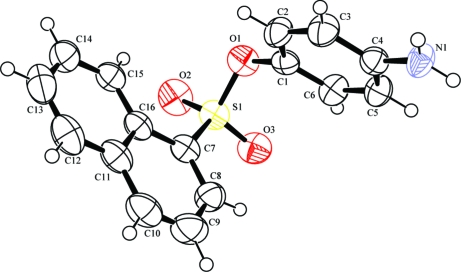
The molecular structure of (I), with atom labels and 50% probability displacement ellipsoids for non-H atoms. Intramolecular H-bonds are shown as dotted lines.

**Fig. 2.**
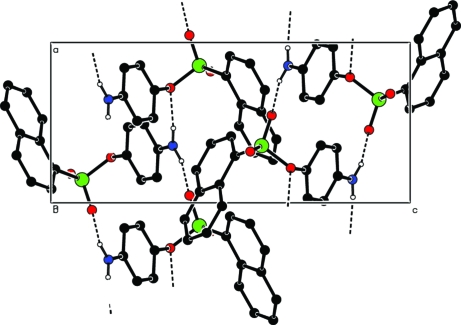
The packing of (I), viewed down the b axis. Hydrogen bonds are shown as dashed lines. H atoms not involved in hydrogen bonding have been omitted.

## supplementary crystallographic information

### Comment

Several compounds containing *para*-toluene sulfonate moiety are used in the fields of biology and industry. The merging of lipids can be monitored using a derivative of *para*-toluene sulfonate (Yachi *et al.*, 1989).

We report the crystal structure of the title compound, (I). The geometric parameters of the molecule of (I) (Fig. 1) agree well with the reported structures (Manivannan *et al.* 2005*a*,*b*). The plane of the aminobenzene ring forms a dihedral angle of 61.04 (6)° with the naphthalene ring.

The molecular structure is stabilized by weak intramolecular C—H···O interactions and the crystal packing is stabilized by weak intermolecular N—H···O interactions and a C—H···π interaction (Table 1), involving the ring C11—C16 (centroid *Cg*).

### Experimental

1-Napthalene sulfonyl chloride (5 mmol) dissolved in acetone (4 ml) was added dropwise to *p*-amino phenol (5 mmol) in aqueous NaOH (4 ml, 5%) with constant shaking. The precipitated compound (3.5 mmol, yield 70%) was recrystlized from ethanol.

### Refinement

H atoms were positioned geometrically (C—H = 0.93 and N—H = 0.86 Å) and refined using riding model, with *U*_iso_(H) = 1.2*U*_eq_(C, N). The anisotropic displacement parameters of O1, S1 and C1 atoms were refined with a rigid bond restraint (DELU) in the final cycles of refinement.

### Crystal data

**Table:**

C_16_H_13_NO_3_S	*F*_000_ = 624
*M**_r_* = 299.33	*D*_x_ = 1.431 Mg m^−^^3^
Orthorhombic, *P*2_1_2_1_2_1_	Mo *K*α radiation λ = 0.71073 Å
Hall symbol: P 2ac 2ab	Cell parameters from 4586 reflections
*a* = 7.0456 (3) Å	θ = 2.8–25.7º
*b* = 12.4789 (6) Å	µ = 0.24 mm^−^^1^
*c* = 15.8064 (8) Å	*T* = 295 (2) K
*V* = 1389.72 (11) Å^3^	Block, brown
*Z* = 4	0.36 × 0.16 × 0.16 mm

### Data collection

**Table:**

Bruker Kappa APEXII diffractometer	4629 independent reflections
Radiation source: fine-focus sealed tube	3267 reflections with *I* > 2σ(*I*)
Monochromator: graphite	*R*_int_ = 0.030
*T* = 295(2) K	θ_max_ = 31.9º
ω and φ scans	θ_min_ = 2.1º
Absorption correction: multi-scan(*SADABS*; Sheldrick, 1996)	*h* = −10→10
*T*_min_ = 0.918, *T*_max_ = 0.962	*k* = −18→18
19808 measured reflections	*l* = −23→19

### Refinement

**Table:**

Refinement on *F*^2^	Hydrogen site location: inferred from neighbouring sites
Least-squares matrix: full	H-atom parameters constrained
*R*[*F*^2^ > 2σ(*F*^2^)] = 0.051	*w* = 1/[σ^2^(*F*_o_^2^) + (0.0956*P*)^2^ + 0.1498*P*] where *P* = (*F*_o_^2^ + 2*F*_c_^2^)/3
*wR*(*F*^2^) = 0.164	(Δ/σ)_max_ < 0.001
*S* = 1.03	Δρ_max_ = 0.44 e Å^−^^3^
4629 reflections	Δρ_min_ = −0.37 e Å^−^^3^
190 parameters	Extinction correction: none
2 restraints	Absolute structure: Flack (1983), 1972 Friedel pairs
Primary atom site location: structure-invariant direct methods	Flack parameter: −0.01 (10)
Secondary atom site location: difference Fourier map	

### Fractional atomic coordinates and isotropic or equivalent isotropic displacement parameters (Å^2^)

**Table:**

	*x*	*y*	*z*	*U*_iso_*/*U*_eq_	
C1	0.2028 (3)	−0.04132 (18)	0.71108 (14)	0.0415 (4)	
C2	0.0284 (3)	−0.0930 (2)	0.71475 (17)	0.0508 (6)	
H2	−0.0763	−0.0641	0.6871	0.061*	
C3	0.0110 (3)	−0.1874 (2)	0.7597 (2)	0.0545 (6)	
H3	−0.1064	−0.2211	0.7637	0.065*	
C4	0.1674 (3)	−0.23186 (18)	0.79873 (15)	0.0443 (5)	
C5	0.3412 (3)	−0.18155 (19)	0.79520 (16)	0.0488 (5)	
H5	0.4463	−0.2117	0.8217	0.059*	
C6	0.3577 (3)	−0.08513 (19)	0.75158 (17)	0.0503 (5)	
H6	0.4740	−0.0500	0.7497	0.060*	
C7	0.2918 (4)	−0.0253 (2)	0.51224 (15)	0.0462 (5)	
C8	0.4146 (4)	−0.1082 (2)	0.5015 (2)	0.0569 (6)	
H8	0.5286	−0.1097	0.5311	0.068*	
C9	0.3689 (6)	−0.1918 (2)	0.4455 (2)	0.0699 (8)	
H9	0.4551	−0.2472	0.4370	0.084*	
C10	0.2039 (6)	−0.1927 (2)	0.40449 (19)	0.0634 (7)	
H10	0.1759	−0.2496	0.3685	0.076*	
C11	0.0689 (4)	−0.1083 (2)	0.41450 (18)	0.0539 (6)	
C12	−0.1042 (5)	−0.1111 (3)	0.3724 (2)	0.0664 (8)	
H12	−0.1329	−0.1691	0.3377	0.080*	
C13	−0.2326 (5)	−0.0302 (3)	0.38116 (19)	0.0685 (8)	
H13	−0.3481	−0.0328	0.3527	0.082*	
C14	−0.1884 (4)	0.0572 (3)	0.43368 (18)	0.0621 (7)	
H14	−0.2748	0.1131	0.4393	0.074*	
C15	−0.0212 (4)	0.0612 (2)	0.47630 (17)	0.0515 (6)	
H15	0.0046	0.1198	0.5108	0.062*	
C16	0.1133 (3)	−0.02101 (18)	0.46937 (15)	0.0451 (5)	
N1	0.1446 (3)	−0.32773 (15)	0.83938 (13)	0.0487 (5)	
H1A	0.2400	−0.3580	0.8635	0.058*	
H1B	0.0348	−0.3578	0.8408	0.058*	
O1	0.2166 (3)	0.05975 (15)	0.66776 (12)	0.0592 (5)	
O2	0.3197 (4)	0.17748 (15)	0.55522 (14)	0.0706 (6)	
O3	0.5453 (3)	0.04850 (19)	0.61441 (14)	0.0659 (6)	
S1	0.35628 (9)	0.07323 (5)	0.58673 (4)	0.04995 (18)	

### Atomic displacement parameters (Å^2^)

**Table:**

	*U*^11^	*U*^22^	*U*^33^	*U*^12^	*U*^13^	*U*^23^
C1	0.0433 (10)	0.0422 (9)	0.0391 (11)	−0.0016 (8)	−0.0002 (9)	−0.0121 (8)
C2	0.0377 (10)	0.0564 (14)	0.0584 (15)	−0.0005 (9)	−0.0021 (10)	0.0099 (12)
C3	0.0381 (10)	0.0611 (15)	0.0644 (16)	−0.0067 (10)	−0.0023 (10)	0.0090 (14)
C4	0.0447 (11)	0.0441 (11)	0.0440 (12)	−0.0009 (9)	0.0012 (9)	0.0003 (9)
C5	0.0444 (11)	0.0485 (12)	0.0535 (14)	−0.0027 (10)	−0.0119 (10)	−0.0009 (11)
C6	0.0442 (10)	0.0461 (12)	0.0607 (15)	−0.0082 (10)	−0.0094 (11)	−0.0036 (11)
C7	0.0534 (12)	0.0399 (11)	0.0452 (12)	−0.0093 (9)	0.0063 (10)	−0.0008 (10)
C8	0.0586 (14)	0.0531 (14)	0.0591 (17)	0.0050 (11)	0.0030 (13)	0.0016 (12)
C9	0.093 (2)	0.0475 (14)	0.0694 (19)	0.0115 (15)	0.0126 (18)	−0.0080 (14)
C10	0.088 (2)	0.0484 (14)	0.0536 (15)	−0.0082 (13)	0.0071 (15)	−0.0097 (13)
C11	0.0719 (15)	0.0475 (13)	0.0423 (13)	−0.0153 (11)	0.0063 (12)	−0.0005 (11)
C12	0.083 (2)	0.0687 (18)	0.0474 (15)	−0.0234 (16)	−0.0050 (14)	−0.0016 (14)
C13	0.0637 (17)	0.092 (2)	0.0500 (15)	−0.0171 (16)	−0.0091 (13)	0.0141 (17)
C14	0.0585 (14)	0.0730 (19)	0.0547 (15)	0.0032 (13)	0.0028 (12)	0.0103 (14)
C15	0.0569 (13)	0.0520 (14)	0.0456 (13)	−0.0040 (10)	0.0064 (10)	0.0026 (11)
C16	0.0550 (12)	0.0409 (11)	0.0395 (11)	−0.0095 (9)	0.0054 (10)	0.0018 (9)
N1	0.0379 (8)	0.0452 (10)	0.0631 (13)	−0.0036 (8)	−0.0047 (9)	0.0182 (9)
O1	0.0663 (10)	0.0520 (9)	0.0594 (10)	−0.0055 (8)	0.0036 (8)	−0.0032 (8)
O2	0.0897 (16)	0.0419 (10)	0.0803 (14)	−0.0233 (10)	0.0025 (12)	0.0052 (10)
O3	0.0465 (9)	0.0750 (14)	0.0763 (13)	−0.0157 (9)	0.0011 (9)	−0.0054 (11)
S1	0.0510 (3)	0.0416 (3)	0.0573 (4)	−0.0135 (2)	0.0011 (3)	−0.0021 (3)

### Geometric parameters (Å, °)

**Table:**

C1—C6	1.378 (3)	C9—H9	0.9300
C1—C2	1.389 (3)	C10—C11	1.428 (5)
C1—O1	1.438 (3)	C10—H10	0.9300
C2—C3	1.381 (4)	C11—C12	1.390 (4)
C2—H2	0.9300	C11—C16	1.427 (3)
C3—C4	1.380 (3)	C12—C13	1.362 (5)
C3—H3	0.9300	C12—H12	0.9300
C4—N1	1.368 (3)	C13—C14	1.406 (5)
C4—C5	1.377 (3)	C13—H13	0.9300
C5—C6	1.392 (3)	C14—C15	1.358 (4)
C5—H5	0.9300	C14—H14	0.9300
C6—H6	0.9300	C15—C16	1.401 (4)
C7—C8	1.360 (4)	C15—H15	0.9300
C7—C16	1.430 (3)	N1—H1A	0.8600
C7—S1	1.762 (3)	N1—H1B	0.8600
C8—C9	1.404 (4)	O2—S1	1.417 (2)
C8—H8	0.9300	O3—S1	1.436 (2)
C9—C10	1.332 (5)	O1—S1	1.624 (2)
			
C6—C1—C2	119.8 (2)	C11—C10—H10	119.3
C6—C1—O1	121.0 (2)	C12—C11—C16	120.2 (3)
C2—C1—O1	119.1 (2)	C12—C11—C10	120.8 (3)
C3—C2—C1	119.7 (2)	C16—C11—C10	119.0 (3)
C3—C2—H2	120.1	C13—C12—C11	121.0 (3)
C1—C2—H2	120.1	C13—C12—H12	119.5
C4—C3—C2	120.1 (2)	C11—C12—H12	119.5
C4—C3—H3	119.9	C12—C13—C14	119.2 (3)
C2—C3—H3	119.9	C12—C13—H13	120.4
N1—C4—C5	121.5 (2)	C14—C13—H13	120.4
N1—C4—C3	117.9 (2)	C15—C14—C13	120.9 (3)
C5—C4—C3	120.6 (2)	C15—C14—H14	119.5
C4—C5—C6	119.2 (2)	C13—C14—H14	119.5
C4—C5—H5	120.4	C14—C15—C16	121.4 (3)
C6—C5—H5	120.4	C14—C15—H15	119.3
C1—C6—C5	120.5 (2)	C16—C15—H15	119.3
C1—C6—H6	119.8	C15—C16—C11	117.3 (2)
C5—C6—H6	119.8	C15—C16—C7	125.8 (2)
C8—C7—C16	121.9 (2)	C11—C16—C7	116.9 (2)
C8—C7—S1	116.8 (2)	C4—N1—H1A	120.0
C16—C7—S1	121.19 (19)	C4—N1—H1B	120.0
C7—C8—C9	119.9 (3)	H1A—N1—H1B	120.0
C7—C8—H8	120.1	C1—O1—S1	120.50 (15)
C9—C8—H8	120.1	O2—S1—O3	118.25 (13)
C10—C9—C8	120.9 (3)	O2—S1—O1	105.20 (13)
C10—C9—H9	119.6	O3—S1—O1	107.43 (12)
C8—C9—H9	119.6	O2—S1—C7	111.02 (13)
C9—C10—C11	121.4 (3)	O3—S1—C7	107.04 (13)
C9—C10—H10	119.3	O1—S1—C7	107.38 (11)
			
C6—C1—C2—C3	0.7 (4)	C14—C15—C16—C11	1.1 (4)
O1—C1—C2—C3	−176.8 (2)	C14—C15—C16—C7	−179.1 (2)
C1—C2—C3—C4	−1.9 (4)	C12—C11—C16—C15	−1.9 (4)
C2—C3—C4—N1	−177.2 (2)	C10—C11—C16—C15	178.9 (2)
C2—C3—C4—C5	1.6 (4)	C12—C11—C16—C7	178.3 (2)
N1—C4—C5—C6	178.7 (2)	C10—C11—C16—C7	−1.0 (3)
C3—C4—C5—C6	−0.1 (4)	C8—C7—C16—C15	−179.7 (3)
C2—C1—C6—C5	0.9 (4)	S1—C7—C16—C15	4.3 (3)
O1—C1—C6—C5	178.3 (2)	C8—C7—C16—C11	0.1 (3)
C4—C5—C6—C1	−1.1 (4)	S1—C7—C16—C11	−175.90 (18)
C16—C7—C8—C9	1.4 (4)	C6—C1—O1—S1	63.1 (3)
S1—C7—C8—C9	177.6 (2)	C2—C1—O1—S1	−119.4 (2)
C7—C8—C9—C10	−2.0 (5)	C1—O1—S1—O2	174.10 (17)
C8—C9—C10—C11	1.2 (5)	C1—O1—S1—O3	−59.1 (2)
C9—C10—C11—C12	−178.9 (3)	C1—O1—S1—C7	55.8 (2)
C9—C10—C11—C16	0.4 (4)	C8—C7—S1—O2	137.0 (2)
C16—C11—C12—C13	1.4 (4)	C16—C7—S1—O2	−46.8 (2)
C10—C11—C12—C13	−179.4 (3)	C8—C7—S1—O3	6.6 (2)
C11—C12—C13—C14	0.0 (4)	C16—C7—S1—O3	−177.21 (18)
C12—C13—C14—C15	−0.8 (4)	C8—C7—S1—O1	−108.5 (2)
C13—C14—C15—C16	0.3 (4)	C16—C7—S1—O1	67.7 (2)

### Hydrogen-bond geometry (Å, °)

**Table:**

*D*—H···*A*	*D*—H	H···*A*	*D*···*A*	*D*—H···*A*
C6—H6···O3	0.93	2.52	3.038 (3)	116
C8—H8···O3	0.93	2.38	2.804 (4)	108
C15—H15···O2	0.93	2.44	3.071 (4)	125
N1—H1B···O1^i^	0.86	2.05	2.909 (3)	173
N1—H1A···O3^ii^	0.86	1.94	2.773 (3)	162
C6—H6···Cg^iii^	0.93	2.84	3.380 (3)	107

Symmetry codes: (i) −*x*, *y*−1/2, −*z*+3/2; (ii) −*x*+1, *y*−1/2, −*z*+3/2; (iii) −*x*+1/2, −*y*, *z*+1/2.

## References

[bb1] Bruker (2004). *APEX2* Bruker AXS Inc., Madison, Wisconsin, USA.

[bb2] Flack, H. D. (1983). *Acta Cryst.* A**39**, 876–881.

[bb3] Manivannan, V., Vembu, N., Nallu, M., Sivakumar, K. & Fronczek, F. R. (2005*a*). *Acta Cryst.* E**61**, o528–o530.

[bb4] Manivannan, V., Vembu, N., Nallu, M., Sivakumar, K. & Fronczek, F. R. (2005*b*). *Acta Cryst.* E**61**, o242–o244.

[bb5] Sheldrick, G. M. (1996). *SADABS*, University of Göttingen, Germany.

[bb6] Sheldrick, G. M. (2008). *Acta Cryst.* A**64**, 112–122. 10.1107/S0108767307043930 18156677

[bb7] Spek, A. L. (2003). *J. Appl. Cryst.* **36**, 7–13.

[bb8] Yachi, K., Sugiyama, Y., Sawada, Y., Iga, T., Ikeda, Y., Toda, G. & Hananon, M. (1989). *Biochim. Biophys. Acta*, **978**, 1–7. 10.1016/0005-2736(89)90490-2 2914125

